# Innate immunity and inflammation in ageing: a key for understanding age-related diseases

**DOI:** 10.1186/1742-4933-2-8

**Published:** 2005-05-18

**Authors:** Federico Licastro, Giuseppina Candore, Domenico Lio, Elisa Porcellini, Giuseppina Colonna-Romano, Claudio Franceschi, Calogero Caruso

**Affiliations:** 1 Dipartimento di Patologia Sperimentale, Università di Bologna, Italy; 2 Gruppo di Studio sull'Immunosenescenza, Dipartimento di Biopatologia e Metodologie Biomediche, Università di Palermo, Italy; 3 Istituto Nazionale di Riposo e Cura per Anziani, Ancona, Italy; 4 Centro Interdipartimentale "L. Galvani", Università di Bologna, Bologna, Italy

**Keywords:** Age-related diseases, Cytokine, Inflammation, Innate Immunity, Longevity

## Abstract

The process of maintaining life for the individual is a constant struggle to preserve his/her integrity. This can come at a price when immunity is involved, namely systemic inflammation. Inflammation is not per se a negative phenomenon: it is the response of the immune system to the invasion of viruses or bacteria and other pathogens. During evolution the human organism was set to live 40 or 50 years; today, however, the immune system must remain active for much a longer time. This very long activity leads to a chronic inflammation that slowly but inexorably damages one or several organs: this is a typical phenomenon linked to ageing and it is considered the major risk factor for age-related chronic diseases. Alzheimer's disease, atherosclerosis, diabetes and even sarcopenia and cancer, just to mention a few – have an important inflammatory component, though disease progression seems also dependent on the genetic background of individuals. Emerging evidence suggests that pro-inflammatory genotypes are related to unsuccessful ageing, and, reciprocally, controlling inflammatory status may allow a better chance of successful ageing. In other words, age-related diseases are "the price we pay" for a life-long active immune system: this system has also the potential to harm us later, as its fine tuning becomes compromised. Our immune system has evolved to control pathogens, so pro-inflammatory responses are likely to be evolutionarily programmed to resist fatal infections with pathogens aggressively. Thus, inflammatory genotypes are an important and necessary part of the normal host responses to pathogens in early life, but the overproduction of inflammatory molecules might also cause immune-related inflammatory diseases and eventually death later. Therefore, low responder genotypes involved in regulation of innate defence mechanisms, might better control inflammatory responses and age-related disease development, resulting in an increased chance of long life survival in a "permissive" environment with reduced pathogen load, medical care and increased quality of life.

## Innate immune system

The first line of immune defence mainly operates by detection of a broad range of molecular patterns foreign to mammalian tissues, called pathogen-associated molecular patterns that induce the activation of the innate immunity and inflammatory response [1]. The constitutive expression of a limited set of pattern recognition receptors by many cell types of the innate immunity does not require clonal expansion of specific cell populations. These germ cell-encoded proteins recognize microbial pathogens or ligands from damaged tissues based on shared molecular structures and induce host responses that localize the spread of infection and enhance systemic resistance to infection. Therefore, the expression of a limited number of highly active genes during the activation of innate immunity is able to induce rapid (minutes to hours) efficient defensive immune responses [2].

Several cell types contribute to innate immunity and the mononuclear phagocyte lineage plays a pivotal role in innate immunity. Monocytes, macrophages and their tissue-differentiated derivatives, such as microglia in the nervous system, express pattern recognition receptors, namely various scavenger and Toll-like receptors [3]. These receptors induce transmembrane signals that activate NF-kB and mitogen dependent protein kinase pathways [4]. Toll-like receptor activation also induces the expression of a wide number of genes encoding proteins, such as cytokines, with regulatory functions upon leukocyte activation and tissue inflammation [5]. Therefore, the capacity of each individual organism to regulate the activation of innate immunity and local inflammatory responses is crucial for initiating defensive action against pathogens, limiting tissue damage and enhancing fast recovery and tissue healing.

## The inflammatory response

In response to cell injury elicited by trauma or infection the inflammatory response sets in, constituting a complex network of molecular and cellular interactions directed to facilitate a return to physiological homeostasis and tissue repair. The response is composed of both local events and a systemic activation mediated by cytokines. If tissue health is not restored or in response to stable low grade irritation, inflammation becomes a chronic condition that continuously damages the surrounding tissues. In fact, during chronic inflammatory immune responses, tissue injury and healing proceed simultaneously. The collateral damage caused by this type of inflammation usually accumulates slowly, sometimes asymptomatically for years but can eventually lead to severe tissue deterioration [6].

Cytokines are largely secreted molecules that act on the surrounding microenvironment by providing cell to cell signalling. Cytokines are components of a large, complex signalling network. The effects of cytokines on target cells may be inhibited or enhanced by other cytokines, hormones, and cytokine-receptor antagonists and circulating receptors. Tumor necrosis factor α (TNF-α), Interleukin-1 (IL-1) and IL-6 are the classical pro-inflammatory cytokines. Their ability to activate both local and systemic effects is well established. Locally, they contribute to the activation of the inflammatory cells and together with chemokines, which induce the expression of adhesion molecules, cause their local recruitment. When the causes of the inflammatory reaction are of a high intensity, the production of cytokines is increased and they are released in the circulation provoking the "acute phase response". On the other hand, "inhibitory" cytokines such as IL-10 damp down the activation of some effector functions of T lymphocytes and mononuclear phagocytes, by inhibiting the release of pro-inflammatory cytokines and therefore turning off the inflammatory processes [7,8].

The acute phase response e.g. leukocytosis, fever, somnelence, anorexia and acute phase proteins synthesis, such as C reactive protein (CRP) in the liver, is a highly conserved inflammatory response which is rapidly activated by infections or trauma via pattern recognition molecules. Acute phase protein concentration rapidly increases after infection, and their production is controlled primarily by IL-6- and IL-1-type cytokines. The acute phase proteins provide enhanced protection against microorganisms and modify inflammatory responses by affecting cell trafficking and mediator release. The more traditional view of acute phase proteins has extended beyond that of opsonization of microorganisms. Some acute phase proteins have anti-inflammatory effects while others have important effects on leukocyte activation and trafficking [9].

CRP, named for its capacity to precipitate the somatic C-polysaccharide of Streptococcus pneumoniae, was the first acute-phase protein to be described, and is an exquisitely sensitive systemic marker of inflammation and tissue damage. It is a member of the pentraxin family of plasma proteins, which are part of the lectin fold superfamily of calcium-dependent ligand-binding and lectin (carbohydrate-binding) proteins. In healthy blood donors, the median concentration of CRP is 0.8 mg/l, but following an acute-phase stimulus, values may increase 10 000-fold. In an apparently healthy population the median baseline value is slightly higher and tends to increase with age with females showing slightly higher circulating concentrations. In most, but not all diseases, the circulating value of CRP reflects on-going inflammation much more accurately than do other biochemical parameters of inflammation, such as plasma viscosity or the erythrocyte sedimentation rate [10].

## Innate immune system during ageing

Age related changes in clonotypic immune system are well documented. For instance, involution of the thymus gland is an early feature of the immune reconfiguration and several age- related alterations of T or B cell compartments have been described [11-13]. Studies on age-associated changes of the innate immunity are not as advanced as those of the clonotypic immune system. However, investigations from aged mice showed a functional decline of monocytes and macrophages, a low expression level of Toll-like receptors from activated splenic and peritoneal macrophages and an altered secretion of several chemokines and cytokines [14,15]. Aged macrophages also contribute to an impaired the proliferative response of activated peripheral T lymphocytes [16].

Aged phagocytes, such as macrophages and neutrophils, showed an impaired respiratory burst and reactive nitrogen intermediate production with a decreased ability to destroy pathogens. Moreover, aged dendritic cells were less efficient in activating both T and B cell populations and aged NK cells showed a decreased ability in killing tumour cells [17,18].

However, not all immune activities are decreased during ageing. In fact, the 'in vitro' production of pro-inflammatory cytokines, such as IL-1, IL-6 and TNF-α, by mitogen activated peripheral blood monuclear cells from elderly persons was higher than that from young donors [19]. Cytokine production may also be up-regulated 'in vivo' in old subjects resulting in an abnormal elevation of pro-inflammatory cytokines during inflammatory responses [20]. An age- related increase of IL-6 levels has also been reported in plasma, serum and supernatants from healthy elderly and centenarians [19,21,22]. It is interesting to note that 'in vitro' addition of IL-1 and TNF-α to fibroblasts induced an accelerated senescent phenotype which was rescued by anti-oxidant addition [23].

## Inflammatory responses during ageing

A dramatic increase in mean life span and life expectancy, coupled with a significant reduction in early mortality, has lead to a large increase in the number of elderly people in modern societies. This demographic phenomenon has been paralleled by an epidemic of chronic disease usually associated with advancing age [24]. Most age-related diseases have complex aetiology and pathogenic mechanisms. The clinical diagnosis and therapy of these diseases requires a multidisciplinary medical approach with progressively increased costs. A body of experimental and clinical evidence suggests that the immune system is implicated, with a variable degree of importance, in almost all age-related or associated diseases. Both innate and the clonotypic immune system are usually involved in the pathogenesis of these chronic diseases [11,25]. However, inflammatory responses appear to be the prevalent triggering mechanism driving tissue damage associated with different age-related diseases and the term "Inflammaging" has been coined to explain the underlining inflammatory changes common to most age-associated diseases [26]. This new term indicates that ageing is accompanied by an age-dependent up-regulation of the inflammatory response, due to the chronic antigenic stress which bombards the innate immune system thorough out life and potentially triggers the onset of inflammatory disease.

Chronic inflammation is considered to be involved in the pathogenesis of all age-related diseases: Alzheimer's disease, atherosclerosis, diabetes, sarcopenia and cancer all have important inflammatory components. Inflammaging, i.e. the up-regulation of a variety of anti-stress responses at the cellular and molecular level, is the consequence of the body's ability to counteract and modulate the effects of a variety of stressors, which cause the accumulation of molecular and cellular scars [26,27]. However, as recently discussed [28], a wide range of different aetiological factors is likely to contribute to increased low-grade inflammatory activity in elderly populations including a decreased production of sex steroids, smoking, subclinical disorders such as arteriosclerosis, asymptomatic bacteruria and a higher relative/absolute amount of fat tissue, which has been suggested to produce pro-inflammatory cytokines [29] (see below). Furthermore, increased levels of circulating inflammatory mediators may result from a constant, low-grade activation of cytokine-producing cells or a dysregulated cytokine response following stimulation [28], which does not readily become damped down. On the other hand, a recent hypothesis suggests that the reduction in lifetime exposure to infectious diseases and other sources of inflammation – the cohort mechanism – may also contribute to the historical decline in old-age mortality [30].

Recent studies have linked an individual's exposure to past infection to levels of chronic inflammation and to increased risk of heart attack, stroke, and cancer. For example, the risk of heart attack is correlated with serum levels of inflammatory proteins such as CRP [31]. Within individuals, CRP levels are also correlated with the number of seropositivities to common pathogens, suggestive of infection history [32].

Low-grade increases in levels of circulating TNF-α, IL-6, soluble IL-2 receptor (sIL-2R), and CRP and low levels of albumin and cholesterol, which also act as inflammatory markers, are strong predictors of all-cause mortality risk in several longitudinal studies of elderly cohorts. The effects of inflammatory mediators upon survival are independent of pre-existing morbidity and other traditional risk factors for death (smoking, blood pressure, physical exercise, total cholesterol, co-morbidity, body mass index, and intake of anti-inflammatory drugs) suggesting that cytokines may induce exaggerated pathological processes. Furthermore these molecules appear to be sensitive markers of pre-clinical disorders in elderly populations [28,33-39].

In the following sections we will focus on the role of innate immunity and inflammation in the most common diseases of elderly in order to direct the reader's interest to these emerging topics in modern gerontology and geriatrics investigations.

## Frailty and sarcopenia

Frailty has been defined as an age-related decline in lean body mass, decreased muscle strength, endurance, balance and walking performance, low activity and weight loss accompanied by a high risk of disability, falls, hospitalisation and mortality [28,40]. It has been suggested that this syndrome reflects a metabolic imbalance caused by overproduction of catabolic cytokines and by the diminished availability or action of anabolic hormones, resulting from ageing itself and the presence of associated chronic conditions [28,41]. Sarcopenia is obviously a central part of the frailty syndrome [28]. Sarcopenia is the loss of muscle mass and strength that occurs with normal ageing. In elderly, there is a decline in a variety of neural, hormonal, and environmental trophic signals to muscle. However, the most important endogenous cause may be the irreversible age-related loss of α-motor units in the central nervous system [42-44]. The most important environmental cause of sarcopenia is lack of physical activity which is an age-related phenomenon which shows universal decline with age in industrialized societies; obesity and type 2 diabetes being concomitant epidemics (see below). An emerging issue in the past few years for sarcopenia research has been whether there is also an increased catabolic signal, driven by systemic inflammation [44]. Systemic low-grade inflammation has been associated with decreased muscle mass as well as the development of functional disability in elderly populations [33,44-47]. TNF-α has special effects that may contribute directly to sarcopenia [44], including increased basal energy expenditure, anorexia, loss of muscle and bone mass 'in vivo' and association with wasting/cachexia in chronic inflammatory disorders. Consistent with these findings, muscle protein synthesis was inversely related to local levels of TNF-α protein in skeletal muscles in frail very old humans [48]. In a recent study of nursing home residents aged 85–96 years, systemic low-grade activation of the TNF-α system at baseline was inversely correlated to muscle strength after resistance training for 12 weeks, demonstrating that TNF-α could also be a limiting factor for training-induced improvement in muscle strength in very old people [49]. The role of IL-6 in sarcopenia is not clear. Epidemiological studies have reported that IL-6 is strongly associated with functional disability and loss of muscle mass but experimental studies have not been able to link IL-6 to sarcopenia [28,44]. Human investigations have shown that inflammation impairs muscle strength in elderly people [50] while Insulin Growth Factor 1 and IL-6 plasma levels were synergistically related to disability and mortality in older women [51].

A complicating factor in untangling the cause-effect relationships underlying sarcopenia is the ability of IL-6, TNF-α, physical inactivity, abdominal obesity, and other factors to cause insulin resistance in the elderly [52-54]. Loss of control of the anabolic action of insulin on muscle would be another factor favouring sarcopenia, resulting in the elevation of one or more catabolic signals. Thus, the weight gain that occurs in most adults during middle age may actually predispose to sarcopenia as they age [44].

## Obesity, the metabolic syndrome, type II diabetes

Western societies face a health care crisis of epidemic proportion, since the number of people with the metabolic syndrome is rising exponentially. This condition constitutes a major challenge for public health professionals in the field of preventive medicine and estimates suggest that more than 40 million US adults will be affected by the syndrome [55].

There is not known survival advantage of morbid obesity; on the other hand increased body fat is linked to high mortality [56]. In the past, humans have been plagued by famine when survival advantage would have been conferred by genes favouring an available energy source: the so called 'thrifty gene' hypothesis. These genes led to genetic selection toward insulin resistance in the peripheral tissue in order to preserve glucose supply of the brain during starvation [57]. Human obesity shows a clear genetic component, which is usually polygenic and polymorphic variances in a number of 'thrifty' genes could contribute to different susceptibilities to obesity and diabetes [58,59].

Metabolic syndrome may be detected by 5 clinical diagnostic criteria 1) abdominal adiposity, 2) hypertriglyceridemia, 3) low high density lipoprotein, 4) hypertension, 5) fasting hyperglycaemia [55].

An involvement of inflammation with pathogenic mechanisms influencing the development of the metabolic syndrome has been suggested [55,60,61] and elevated CRP, IL-6 and TNF-α, associated with visceral adiposity, have been reported in this syndrome [61]. Adipocytes constitutively express the pro-inflammatory TNF-α [54] which decreases after weight loss [62]. Further work in this area has confirmed increased plasma concentrations of CRP, IL-6, and plasminogen activator inhibitor-1 (PAI-1) [63-65]. Therefore, a complex interplay may exist between inflammatory responses and general metabolism in atherosclerosis, cardiovascular disease, metabolic syndrome.

It is becoming clear that the increased adipose tissue is not a simple reservoir for excess nutrients, but rather an active and dynamic organ capable of expressing several cytokines and other fat-derived peptides (FDP). Some FDP may have a role in the development of the metabolic syndrome but there is no evidence that these FDP are directly causing inflammation. It has been suggested that high levels of inflammatory factors are markers for obesity/abdominal obesity seen with aging, but some of these may not necessarily have a causative role in the development of inflammation [29]. Alternatively, a positive correlation was found between the body mass index and the percentage of resident macrophages, suggesting that fat tissue growth is associated with a recruitment of blood monocytes, responsible for cytokine production [66].

Insulin resistance is due to the reduced ability of peripheral tissues to properly respond to the activation induced by insulin. It is a key feature in the pathogenesis of type II diabetes and this condition may precede by 10–20 years the onset of hyperglycaemia and the clinical manifestation of the disease. Recent data suggests that a defect in insulin activation of glucose transportation in muscle cells could be induced by serine kinase cascade activation, which is down stream mediators of tissue inflammation factors [67].

Data from the Insulin Resistance Atherosclerosis Study showed that insulin resistance, as assessed by frequently sampled glucose tolerance tests, correlated with high blood levels of CRP, fibrinogen and PAI-1 and levels of these inflammatory factors were predictors of type II diabetes development [68]. Increased blood concentrations of TNF-α and IL-6 were associated with obesity and type II diabetes [69]. Finally, results of the population study from the European Prospective Investigation into Cancer and Nutrition Potsdam indicated a significant interaction between plasma IL-1β, IL-6 and type II diabetes development. In fact subjects with detectable IL-1β levels and increased levels of IL-6 showed an independently elevated risk of developing the disease [70].

Inflammation may predispose to a pre-diabetic state by increasing insulin resistance, since pre-diabetic subjects showed increased plasma levels of inflammatory proteins without primary defects of beta cell functions [71]. Sub-clinical inflammation was found significantly related to insulin resistance in a high risk group for diabetes, i.e. in subjects with positive family history of diabetes, obesity and hyper or dyslipoproteinemia [72]. Recent results from the INCHIANTI population study showed that subjects in the upper tertile of insulin resistance had increased serum levels of TNF-α, IL-1R antagonist and IL-6 and low levels of sIL-6R [73].

TNF-α causes an inhibition of auto-phosphorylation of tyrosine residues of the insulin receptor (IR) and an induction of serine phosphorylation of insulin receptor substrate-1, which in turn causes serine phosphorylation of the IR in adipocytes and inhibits tyrosine phosphorylation [74]. More recently, IL-6 has been shown to inhibit insulin signal transduction in hepatocytes [53]. Therefore, cytokines show relevant metabolic effects.

Novel data have now appeared showing that the concomitant presence of the promoter polymorphisms of TNF-α and IL-6, linked to high production of these cytokines increases the risk of conversion to type 2 diabetes in obese subjects with impaired glucose tolerance response [75].

As discussed by Dandona et al. [69], two mechanisms might be involved in the pathogenesis of inflammation. Glucose and macronutrient intake causes oxidative stress and inflammatory changes. Chronic overnutrition (obesity) might thus be a pro-inflammatory state with oxidative stress. The increased concentrations of TNF-α and IL-6, associated with obesity and type 2 diabetes, might interfere with insulin action by suppressing insulin signal transduction, which in turn might promote inflammation. In fact, insulin reduces ROS generation by mononuclear cells, suppresses NADPH oxidase expression and intranuclear NF-kB binding, induces IkB expression and suppresses some inflammatory molecules [69,76].

The well-known beneficial effects of caloric restriction on longevity in animal models by inducing reduced visceral fat mass might also induce a reduced secretion of multiple metabolically active factors, which are potentially responsible for the development of insulin resistance. This decrease in fat mass and its beneficial effects observed in ageing animal models might apply also to human ageing and its related pathology [77].

## Cancer diseases

The majority of cancer occurs in subjects over the age of 65 years. Cancer rates increase sharply with age in both sexes: the incidence of cancer is 12–36 times higher in individuals aged 65 years or older compared with individuals aged 25–44 years, and 2–3 times more common than in persons aged 45–64 years. It is worth noting that 70% of deaths attributable to all cancer occur in men and in women aged 65 years or older whereas 35% cancer deaths in men and 46% of cancer deaths in women occur in those aged 75 years or older. The relationship between ageing and cancer is similar for most cancers, and it is well described by the multistage model. So, ageing might be considered not as a determinant of cancer per se, but as a surrogate marker of the duration of exposure to relevant carcinogenic factors [78-80].

Chronic inflammation induced by either biological, chemical, mechanical or physical injuries has been associated with increased incidence of cancer in different human tissues [81]. For instance, inflammatory bowel disease, ulcerative colitis and Crohn's disease are clinical conditions predisposing to cancer development of the large bowel or terminal ileum [81,82].

Helicobacter pylori microorganisms are associated with atrophic gastritis, mucosal dysplasia, gastric adenocarcinoma and an unusual form of gastric lymphoma [81,82]. Schistosome and trematode infections have been associated with cancers of the bladder and the biliary tracts [81].

The recognition of a role for inflammation in the natural history of a tumor has been known stretching from the mid-19th century [83]. The inflammatory microenvironment of tumors is characterised by the presence of host leucocytes both in the supporting stroma and in the tumor area [84]. The question whether the inflammatory infiltrate helps or hinders tumors is still open. In fact, inflammatory cells and cytokines found in tumors can contribute to tumor growth, progression, and immunosuppression as well mounting an effective host anti-tumor response. However, in most cases inflammation plays a role in the development of solid cancer. That is clearly demonstrated by a prospective, nested case-control study of a cohort of 22,887 adults followed for 11 years. A total of 172 colorectal cancer cases were identified. Up to 2 controls (n = 342) were selected for each case and matched by age, sex, race, and date of blood draw. Plasma CRP concentrations were higher among all colorectal cases combined compared with controls and the risk of colon cancer was higher in persons in the highest vs. lowest quartile of CRP [85].

Furthermore, in another recent study, a total of 174 patients considered to have undergone curative resection for cancer were studied. The results show that raised circulating concentrations of CRP, whether measured before or after operation, predict overall and cancer-specific survival in patients undergoing potentially curative surgery for colorectal cancer suggesting that the presence of a systemic inflammatory response predicts a poor outcome [86]. This data supports the hypothesis that inflammation is a risk factor for the development of some kind of solid tumors such as colon cancer.

Cancer susceptibility and severity may also be associated with functional polymorphisms of cytokine genes involved in regulation of inflammation. In particular, as discussed in a recent review [79], a considerable body of data indicates that particular cytokine polymorphisms, especially those involving IL-6 and IL-10 genes, may influence susceptibility to, and in some cases prognosis in neoplastic diseases. It is intriguing that these two cytokines are involved in longevity [87-89]. Discrepant results might depend on confounding factors that affect case-control studies [90] or as discussed by Caruso et al. [79] might also depend on pleiotropic action of cytokines. It is paradigmatic in this respect that the favourable effect of high producer IL-10 genotype on hepatocarcinoma induced by HBV [91], may be due to the relative lack of control of HBV infection by immunosuppressive effects of high IL-10 levels.

On the other hand, several and diverse mechanisms may link inflammation to cancer.

1) Free radical production, derived by oxygen or NO metabolic pathways; the respiratory burst of leukocytes, and the arachidonic acid cascade activation are strongly implicated in DNA modification and protein damage which in turn promotes carcinogenesis [92].

2) High constitutive hyper-expression of NF-kB, an ubiquitous transcription factor with regulatory effect upon different inflammatory, apoptotic and oncogenic genes [82].

3) Alterations of the p53 gene induced by NO derived radicals promoting clonal expansion of aberrant or mutated cells [92].

4) Induction of angiogenesis by inflammatory factors favouring cancer progression [92].

5) Increased release of key pro-inflammatory factors and some cytokines, such as IL-1β, TNF-α and interferon (IFN)-γ, implicated in both the regulation of inflammation and the development of cancer [92,93].

## Atherosclerosis and cardiovascular diseases

Atherosclerosis and its complications are a major problem

contributing to large sections of morbidity and mortality in old people. Cardiovascular disease is the leading worldwide cause of morbidity and death in Western societies [94]. However, our understanding of pathogenic mechanisms underling atherosclerosis and its complications is still incomplete, since more than half of patients with atherosclerosis do not show classical risk factors, such as hypercholesterolemia, hypertension, history of smoking, diabetes, obesity and sedentary life style [94,95].

**Figure 1:**
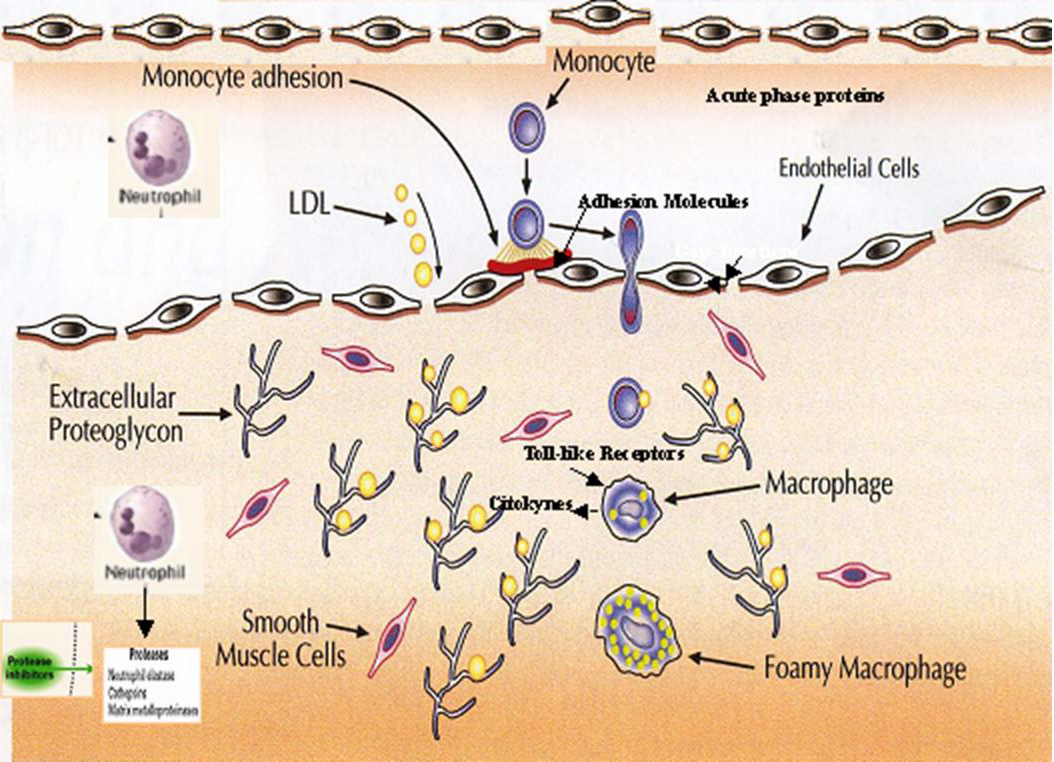
Schematic representation of inflammatory mechanisms involved in pathogenesis of atherosclerosis and plaque formation. Monocytes and macrophages are the protagonists of atherosclerotic processes.

On the other hand, atherosclerosis, formerly considered an inocuous lipid storage disease, actually involves an ongoing inflammatory response (Figure 1). Recent advances in basic science have established a fundamental role for innate immunity in mediating all stages of this disease from initiation through progression and, ultimately, the thrombotic complications of atherosclerosis [96-98]. Clinical studies have shown that the emerging biology of inflammation in atherosclerosis applies directly to human patients. Elevation in markers of inflammation predicts outcomes of patients with acute coronary syndromes, independently of myocardial damage. In addition, low-grade chronic inflammation, as indicated by levels of the inflammatory marker CRP and cytokines, prospectively defines risk of atherosclerotic complications, thus adding to prognostic information provided by traditional risk factors. In fact, levels of CRP or IL-6 have been suggested as significant predictive risk factors for future development of cardiovascular events [31,95,99-103]. Increased levels of serum IL-1β have also been associated with high risk of congestive heart failure and angina pectoris [104]. Altered level of IL-1β is also suggested to be implicated in chronic inflammation underlining high blood pressure [105].

An initiating event is the accumulation of lipids in the vessel wall, which subsequently will become modified and triggers an inflammatory process. Low-density lipoproteins (LDL) are taken up by macrophages through scavenger receptors, leading to foam cells; the lipid deposits become oxidized forming pro-inflammatory lipid peroxides. Monocytes are attracted from the blood and differentiate into macrophages that take up the modified LDL and form lipid-laden foam cells, which is the first hallmark of atherosclerotic plaque development. Later on, inflammatory mediators increase, other immune cells are attracted, and smooth muscle cells are activated and become involved. More advanced stages of plaque development are characterized by increased deposition of extra cellular lipid cores, fibrous material, and often necrosis. Subsequently, these macrophages are further activated, leading to the production of a wide range of cytokines and growth factors. Myocardial infarction may occur as a result of erosion or uneven thinning and rupture of the fibrous cap, often at the shoulders of the lesion where macrophages enter, accumulate and are activated and where apoptosis may occur [7,96-98].

Because genetic traits contribute significantly to the risk of coronary heart disease [106], a number of studies have now addressed the hypothesis that allelic variations in genes of innate immunity may increase the risk of this disease [107,108]. Differences in the genetic regulation of inflammatory processes might partially explain why some people, but not others, develop the disease and why some develop a greater inflammatory response than others. Accordingly, common gene polymorphisms controlling high production of inflammatory molecules have been associated with atherosclerosis and a good control of inflammation might play a protective role against atherosclerosis [107-110]. Recently, a combination of alleles in different inflammatory genes has been associated with increased risk of developing acute myocardial infarction in elderly men [111].

In addition to innate immunity, clonotypic immunity plays also a role in atherosclerosis. Recent findings support the hypothesis that a crucial component of atherosclerosis is represented by T cell-mediated immune responses that are inappropriate in terms of time of onset, intensity, and target [112]. On the other hand, the most direct evidence for the critical role for T cells, IFN-γ, and IFN-γ-driven molecules in atherosclerosis is provided by mice with combined deficiencies of apolipoprotein E (apoE) and the IFN-γ receptor, in which the development of atheroma is significantly reduced in comparison to mice with only apoE deficiency, whereas exogenous IFN-γ enhances atherosclerosis [113,114]. In contrast, the role of B cells has remained unclear. B-cells are not always present and may be protective. In fact, recent studies suggest that this cell type may inhibitthe development of vascular pathology in models of atherosclerosisand restenosis [115]. Finally, Wick et al. [116] have developed an *autoimmune-inflammatory *concept of atherogenesis based on experimental and clinical models. They argue that atherosclerosis may be the price humans pay for pre-existing immunity to microbial or autologous heat shock protein (HSP) 60. In summary, their autoimmune hypothesis of atherogenesis postulates that HSP60 expression is induced in arterial endothelial cells as a response to the action of stress factors, notably the classical atherosclerotic risk factors. Autologous HSP60 epitopes exposed on the surface of stressed endothelial cells are recognized by either pre-existing humoral and cellular anti-microbial HSP60 immune reactions or by invoking *bona fide *autoimmunity based on exposure of altered autologous HSP60 epitopes. The authors accept the importance of well-established atherosclerotic risk factors during atherogenesis, but assign a new role for them in the earliest stages of the disease, viz. acting as stressors [116].

## Brain degenerative diseases: Alzheimer's disease

Alzheimer's disease (AD), a heterogeneous and progressive neurodegenerative disease which in Western societies mainly accounts for clinical dementia, is expected in the USA to rise from 4.6 today to 16 millions cases in 2050 [117]. Neuro-pathological hallmarks of AD are neuronal and synapsis loss, extracellular amyloid deposits (neuritic plaques) and intracellular deposition of degenerate filaments (neurofibrillary tangles) [118]. Major clinical manifestations of the disease are memory loss and cognitive impairment [119].

Inflammation clearly occurs in pathologically vulnerable regions of the AD brain, and it does so with the full complexity of local peripheral inflammatory responses. In the periphery, degenerating tissue and the deposition of highly insoluble abnormal materials are classical stimulants of inflammation. Likewise, in the AD brain damaged neurons and neuritis, highly insoluble Aβ42 peptide deposits and neurofibrillary tangles provide obvious stimuli for inflammation. Senile plaques in AD brains are associated with reactive astrocytes and activated microglial cells; cytokines and acute phase proteins are also overexpressed in microglia and astrocytes surrounding neuropathological lesions in AD brains. Inflammatory factors, such as cytokines, chemokines, complement components and acute phase proteins co-localize as secondary components in neuritic or senile plaques, or are over-produced in AD brains. Finally, activated microglia surrounds senile plaques and areas of neurodegeneration [120,121]. There is accumulating evidence that Aβ peptide may promote or exacerbate inflammation by inducing glial cells to release immune mediators. Moreover, microglial and astroglial cells surrounding mature plaques in AD brains have been found to express activation markers. Enriched populations of human microglial cells isolated from mixed cell cultures prepared from embryonic human telencephalon tissues are able to express constitutively mRNA transcripts for cytokines and chemokines and treatment with pro-inflammatory stimuli as lipopolysaccharide or Aβ peptide leads to increased expression of mRNA levels of these inflammatory molecules [122].

The role of inflammation is further emphasized by a number of epidemiological studies demonstrating that the long-term use of nonsteroidal anti-inflammatory drugs may protect against AD. There are now a several published observational studies demonstrating that people who are known to be taking anti-inflammatory drugs considerably reduce their odds of developing AD and population studies have confirmed this negative association [123]. However, alternative hypothesises have been proposed. In particular, this effect has been hypothesised as relating to the ability of these drugs to inhibit angiogenesis. In fact, the brain endothelium secretes the precursor substrate for the β-amyloid plaque and a neurotoxic peptide that selectively kills cortical neurons. So, antiangiogenic drugs targeting the abnormal brain endothelial cell might be able to prevent and treat this disease [124]

The long-term prospective association between dementia and the well known inflammation marker CRP was evaluated in a cohort of Japanese American men. These subjects were seen in the second examination of the Honolulu Heart Program (1968–1970) and subsequently were re-examined 25 years later for dementia in the Honolulu-Asia Aging Study (1991–1996). In a random subsample of 1,050 Honolulu-Asia Aging Study cases and noncases, high-sensitivity CRP concentrations were measured from serum taken at the second examination; dementia was assessed in a clinical examination that included neuroimaging and neuropsychological testing and was evaluated using international criteria. Compared with men in the lowest quartile (<0.34 mg/L) of high-sensitivity CRP, men in the upper three quartiles had a 3-fold significantly increased risk for all dementias, mainly Alzheimer's disease and vascular dementia. These data support the view that inflammatory markers may reflect not only peripheral disease, but also cerebral disease mechanisms related to dementia, and that these processes are measurable long before clinical symptoms appear [125].

On the other hand, several other investigations have shown increased blood levels of some cytokines, such as IL-1β and IL-6, and acute phase proteins α-1-antichymotrypsin, (ACT) in patients with clinical AD [126-129]. Therefore, altered immune responses in the brain and the peripheral blood appeared to be associated with the disease. Finally, plasma levels of ACT also correlated with the degree of cognitive impairment in AD patients from a case-control study [126] suggesting that peripheral markers of inflammation or impaired immune responses could be used for monitoring the progression of the disease.

Elevated levels of IL-6 in both brain homogenates and peripheral blood from AD patients have also been reported [130]. These findings suggest that an important, but still largely unknown, interplay between brain and peripheral immune responses may exist in the disease.

**Figure 2:**
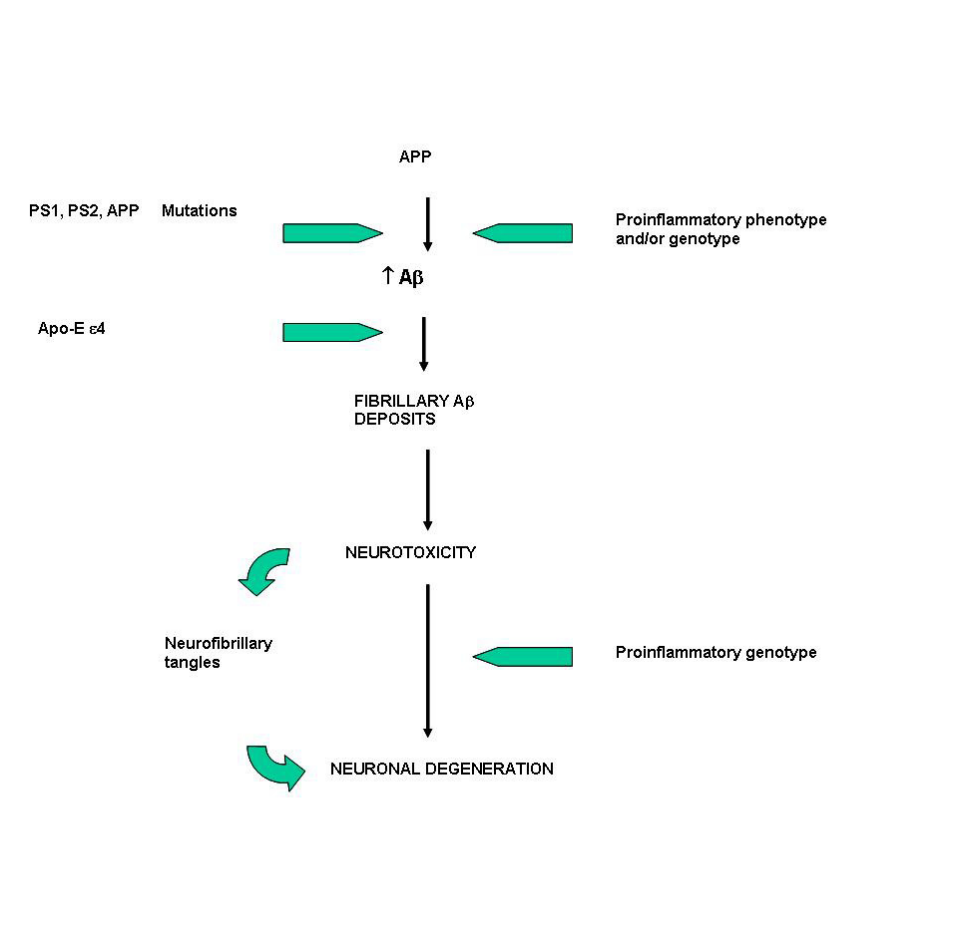
Alzheimer's disease: amyloid deposition is one the main pathogenetic mechanism. Accumulation of Aβ peptide may be caused by 1) gene mutations (PS1, PS2 and APP human mutations in familial Alzheimer's disease) 2) genotype (and/or phenotype) favoring unbalanced inflammatory responses (pro-inflammatory genotype/anti-inflammatory genotype).

In conclusion, the brain lesions associated with AD, which are referred to as neurofibrillary tangles and senile plaques, are characterized by the presence of a broad spectrum of inflammatory mediators, produced by resident brain cells, including neurons. Although secondary to the fundamental pathology caused by the presence of tangles and plaques, there is strong evidence that inflammation exacerbates the neuronal loss. Accordingly, several reports have appeared indicating that the risk of AD is substantially influenced by several polymorphisms in the promoter region, and other untranslated regions, of genes encoding inflammatory mediators. Alleles that favour increased expression of the inflammatory mediators or alleles that favour decreased expression of anti-inflammatory mediators are more frequent in patients with AD than in controls. The polymorphisms are fairly common in the general population, so there is a strong likelihood that any given individual will inherit one or more of the high-risk alleles [121,127,129-135] (Figure 2).

## Conclusion

In this presentation we have presented evidence linking innate immunity with the pathogenesis of age-related chronic degenerative diseases. Inflammation may be a pivotal and common background shared by the majority of these pathological conditions. Although evidence already presented has focused on innate immunity, clonotypic immunity has support as an emerging mechanism related to the pathogenesis of these diseases [116].

The evidence that a decline in mortality exists when comparing Swedish cohorts born in the middle of the 18^th ^century to those in the 20^th ^suggests important links between mortality in old age and in early life. Cohort mortality during childhood has been linked to cohort mortality in old age, implying that early exposure to infection is important in determining and imprinting the cohort morbidity phenotype [30]. Thus, chronic inflammatory mechanisms carry the imprint of early-life infections into later life morbidity and mortality. Evidence presented suggests that serological indicators of infection and inflammation in present day populations are related to vascular disease and other morbidities of ageing. Such inflammatory responses can be induced by invading pathogens, as well as by trauma or internal tissue injury. Thus, adaptive responses to short term infections or injury can become maladaptive as life progresses -a double-edged sword that evolutionary biologists refer to as antagonistic pleiotropy [136].

The process of life for the individual is the struggle to preserve its biological and immunological integrity. However, the preservation of the integrity of the organism comes with the price of responsiveness to systemic inflammation [137] which must be finely tuned otherwise dysregulation becomes a damaging accompanynt. With ageing, the reason why the innate immune system becomes over-activated is not clear, but increased exposure to infectious agents or cumulative damage to tissues could spark the change. Inflammation is not 'per se' a negative phenomenon: it is the response of the immune system to pathogenic viruses or bacteria. Thorough out evolution, man has been set to live about 40 or 50 years but in today's world the immune system is active for several decades compared with the past centuries. A long period of activitation may lead to chronic inflammation which inexorably damages several/all organs and is the phenotype linked to both ageing and chronic disease. In cardiovascular disease, conventional risk factors remain important, but differential baselines in inflammatory status may explain why cholesterol levels seem not always directly associated with cardiovascular disease [22,109].

Low-grade inflammation is also associated with parameters such as obesity, smoking, and physical inactivity, so inflammatory mediators constitute a link between life style factors, infections and physiological changes in the process of ageing on the one hand, and risk factors for age-related diseases on the other [28]. This immune response also depends on the genetic background of individuals. In fact, emerging evidence suggests that polymorphic alleles of inflammatory cytokines, involved in high cytokine production, are related to 'unsuccessful' ageing as noted previously with respect to atherosclerosis and AD. On the other hand, controlling inflammatory status may enhance individual chance of achieving 'successful' ageing. So, major findings reporting a relationship between cytokine polymorphisms and longevity suggest that those individuals who are genetically predisposed to produce low levels of inflammatory cytokines or high levels of anti-inflammatory cytokines may have an increased capacity to reach the extreme limit of human life-span [87-89,109,138,139].

In other words, age-related diseases are "the price we pay" for an active immune system that defends us in youth but may harm us later on [139,140]. Such data support the notion that antagonistic pleiotropy [136] plays a relevant role in diseases and longevity. Pro-inflammatory genotypes may therefore be both friends and foes. They are an important and necessary part of the normal host responses to pathogens, but the overproduction of inflammatory molecules may aggravate immune-inflammatory-related disease and contribute to earlier death. An immune system evolved to control pathogens is likely to favour a highly charged pro-inflammatory response programmed to resist fatal infections with avoidance of early mortality. However, persons with low responder genotypes may risk earlier mortality in response to serious infection but if they survive may respond less aggressively to age-related disease development. Such conditions might result in an increased chance of longevity in an environment with reduced pathogenic antigen load and/or adequate medical treatment [139,141,142].

Further studies on this field will open the way for new diagnostic approaches for early diagnosis of relevant pre-clinical states of age-related diseases. It may be possible, before clinical manifestations appear, that anti-inflammatory or other treatments might play a decisive role in preventing or significantly retarding the manifestation of the disease. Other studies focused on clarifying the specific contribution of each immune factor to a given disease will also contribute to the discovery of new drugs and/or innovative intervention protocols specific for these diseases related to ageing.
